# Supplementary material for: Trade-offs during the COVID-19 pandemic: A discrete choice experiment about policy preferences in Portugal
Source: PLoS One. 2022 Dec 16;17(12):e0278526. doi: 10.1371/journal.pone.0278526 (PMC9757580; doi:10.1371/journal.pone.0278526)
Supplement: S1 Appendix — (PDF) [file pone.0278526.s001.pdf]

Supporting information

Figure S1. DCE instructions given to the respondents (Portuguese)

Instruções para preenchimento:

1. As escolhas deverão ser tomadas assumindo que as consequências escolhidas **persistirão em Portugal durante 6 meses, entre Janeiro e Junho de 2021.**

2. Para efeitos de resposta, por favor considere os seguintes níveis **de restrições à vida quotidiana**:

- Reduzidas (restrições a grandes eventos, bares e discotecas encerradas)
- Intermédias (recolher obrigatório às 23h, restrição forte nos ajuntamentos (5 pessoas))
- Elevadas (limitação à circulação, teletrabalho, recolhimento ao fim-de-semana, serviços não essenciais e escolas encerradas)

3. Considere que não existe causalidade direta entre os impactos apresentados dentro de cada cenário.
Por exemplo: medidas mais restritivas na vida quotidiana não levam necessariamente a menor número de mortes durante o mesmo período, devendo considerar o excesso de mortes que lhe é indicado no cenário.

4. Em três dos domínios são apresentados valores percentuais. Exemplificamos a interpretação de duas possibilidades em baixo:

- 35% da população em risco de pobreza - significa que 35 em cada 100 pessoas estão em risco de pobreza.
- Redução de 20% do rendimento mensal do seu agregado familiar - equivale a uma perda de 200 euros em cada 1000 euros anteriormente recebidos (se recebia 1000 euros passa a receber 800 euros)

Agradecemos imenso a sua participação e o tempo dispensado !

DCE Block 1

Entre as duas opções abaixo descritas, qual seria a sua preferência?

Figure S2. Choice set example, opt-out option and opt-out question (Portuguese)

Para o período Janeiro-Junho 2021	Opção A	Opção B
Excesso de mortes por dia devido à pandemia (direta ou indiretamente)	150 mortes	50 mortes
Quebra do rendimento do seu agregado familiar	10%	30%
População escolar com aprendizagem prejudicada, comprometendo o desempenho futuro	20%	30%
Nível de restrições à vida quotidiana*	Reduzidas	Elevadas
População em risco de pobreza (a viver com <6 euros/dia)	25%	45%

* Restrições Reduzidas (restrições a grandes eventos, bares e discotecas encerradas)
Restrições Intermédias (recolher obrigatório às 23h, restrição forte nos ajuntamentos (5 pessoas))
Restrições Elevadas (limitação à circulação, teletrabalho, recolhimento ao fim-de-semana, serviços não essenciais e escolas encerradas)

Prefiro não continuar a responder ao questionário ○ | OPÇÃO A ○ | OPÇÃO B ○

Porque razão preferiu deixar de responder ao inquérito?

- ☐ A escolha entre as opções é difícil de se fazer
- ☐ Não concordo com o tipo de questões levantadas
- ☐ Não tenho tempo para continuar a responder
- ☐ [ ] Outro

Entre as duas opções abaixo descritas, qual seria a sua preferência?

Table S1. Key descriptive statistics for the sample by gender and working place

	Female	Male	Work from Home	Not Working from Home
	N (%)	N (%)	N (%)	N (%)
**Age**				
19-25 years	448 (32.49)	196 (24.29)	418 (37.29)	228 (21.31)
26-35 years	479 (34.74)	258 (31.97)	301 (26.85)	438 (40.93)
36-45 years	241 (17.48)	184 (22.80)	197 (17.57)	229 (21.40)
46-55 years	131 (9.50)	88 (10.90)	113 (10.08)	106 (9.91)
56-65 years	72 (5.22)	63 (7.81)	74 (6.60)	61 (5.70)
66-75 years	7 (0.51)	14 (1.73)	16 (1.43)	5 (0.47)
76-85 years	1 (0.07)	3 (0.37)	2 (0.18)	2 (0.19)
> 85 years	0 (0.00)	1 (0.12)	0 (0.00)	1 (0.09)
**Gender**				
Female			658 (58.96)	721 (67.38)
Male			458 (41.04)	349 (32.62)
**Education attainment level**				
No college Degree[1]	384 (27.85)	194 (24.07)	316 (38.20)	263 (24.60)
Bachelor's degree[2]	458 (33.21)	253 (31.39)	326 (29.08)	385 (36.01)
Master's Degree or Higher[3]	532 (38.58)	358 (44.42)	474 (42.28)	420 (39.29)
**Monthly equivalent household income**				
< €750	367 (26.61)	129 (15.99)	251 (22.39)	246 (22.99)
€751-€1,250	346 (25.09)	140 (17.35)	247 (22.03)	240 (22.43)
€1,251-€1,750	296 (21.46)	217 (26.89)	271 (24.17)	244 (22.80)
> €1,750	259 (18.78)	259 (32.09)	253 (22.57)	265 (24.77)
No answer	111 (8.05)	62 (7.68)	99 (8.83)	75 (7.01)
**Area of residence**				
Porto	595 (43.18)	319 (39.88)	529 (47.32)	388 (36.43)
Lisbon	302 (21.92)	210 (26.25)	239 (21.38)	273 (25.63)
Other	481 (34.90)	271 (33.87)	350 (31.30)	404 (37.94)
**Number of persons in household**				
1	113 (8.19)	63 (7.81)	89 (7.94)	88 (8.22)
2	394 (28.57)	242 (29.99)	330 (29.44)	307 (28.69)

3	381 (27.63)	202 (25.03)	285 (25.42)	298 (27.85)
4	375 (27.19)	228 (28.25)	318 (28.37)	287 (26.82)
5	97 (7.03)	47 (5.82)	76 (6.78)	69 (6.45)
> 5	19 (1.38)	25 (3.10)	23 (2.05)	21 (1.96)
**Number of children in household**				
Younger than 6	199 (14.90)	86 (11.05)	111 (10.26)	174 (16.78)
Both older and younger than 6	64 (4.79)	37 (4.76)	42 (3.88)	59 (5.69)
Older than 6	273 (20.43)	172 (22.11)	253 (23.38)	193 (18.61)
None	800 (59.88)	483 (62.08)	676 (62.48)	611 (58.92)
**Occupation**				
Unemployed	84 (6.09)	24 (2.97)	45 (3.93)	64 (5.98)
Student	413 (29.95)	205 (25.40)	429 (38.27)	191 (17.85)
Student-Worker	17 (1.23)	4 (0.50)	7 (0.62)	14 (1.31)
Researcher	20 (1.45)	14 (1.73)	31 (2.77)	3 (0.28)
Public servant	303 (21.97)	154 (19.08)	190 (16.95)	267 (24.95)
Retired	13 (0.94)	20 (2.48)	22 (1.96)	11 (1.03)
Large enterprise employee	181 (13.13)	145 (17.97)	155 (13.83)	171 (15.98)
SME employee	236 (17.11)	137 (16.98)	146 (13.02)	229 (21.40)
Self-employed	96 (6.96)	97 (12.02)	84 (7.49)	110 (10.28)
Other	7 (0.51)	2 (0.25)	5 (0.45)	4 (0.37)
**Home Office**				
Yes	658 (47.72)	458 (56.75)		
No	721 (52.28)	349 (43.75)		

1 - Levels 0, 1, 2, and 3 (ISCED)
2 - Level 6 (ISCED)
3 - Level 7 and 8 (ISCED)

Table S2. Coefficients from the main conditional logit model and the respective MRS.

**Choice**	**Coef.**	**95%**	**CI**	**Coef.**	**95%**	**CI**	**MRS**	**95%**	**CI**
Deaths - 150	-1.059	-1.116	-1.003	-	-	-	-	-	-
Deaths - 250	-2.074	-2.167	-1.981	-	-	-	-	-	-
Deaths - continuous	-	-	-	-0.010	-0.011	-0.010	-	-	-
Household income lost - 20%	-0.111	-0.155	-0.066	-0.113	-0.157	-0.069	-10.86	-15.15	-6.58

Household income lost - 30%	-0.490	-0.539	-0.441	-0.486	-0.534	-0.438	-46.81	-51.63	-41.98
Compromised education - 20%	-0.297	-0.339	-0.255	-0.299	-0.341	-0.258	-28.84	-32.80	-24.88
Compromised education - 30%	-0.264	-0.312	-0.216	-0.263	-0.311	-0.215	-25.32	-29.94	-20.70
Life restrictions - medium	-0.195	-0.239	-0.151	-0.197	-0.241	-0.154	-19.02	-23.34	-14.70
Life restrictions - high	-0.240	-0.291	-0.190	-0.244	-0.294	-0.194	-23.54	-28.58	-18.49
Risk of poverty - 35%	-0.426	-0.473	-0.380	-0.425	-0.471	-0.378	-40.90	-45.45	-36.35
Risk of poverty - 45%	-1.049	-1.115	-0.984	-1.050	-1.116	-0.985	-101.17	-107.04	-95.30

The first column of results shows the coefficients of the model treating deaths as a categorical variable.
Number of observations: 35,054; Log likelihood = -9871.67; AIC: 19763.33; BIC: 19847.98
The second column of results shows the coefficients of the model treating deaths as a continuous variable.
Number of observations: 35,054; Log likelihood = -9872.1979; AIC: 19762.40; BIC: 19838.58
The third column of results shows the marginal rate of substitution computed from the second column of results using deaths as the numeraire.

Table S3. MRS from the conditional logit model with deaths modelled as a continuous attribute - Subgroups.

	Income lost		Education lost		Life Restrictions		Poverty		N
	2	3	2	3	2	3	2	3	
Gender									
Female	-6.88	-42.96	-25.42	-18.23	-12.53	-8.45	-39.82	-101.50	22,064
Male	-17.02	-55.35	-35.02	-40.24	-31.68	-53.46	-42.76	-101.50	12,901
Age group									
<25	-14.79	-52.87	-28.04	-32.30	-17.51	-19.09	-49.10	-105.05	10,336
26-45	-6.99	-44.01	-28.39	-24.14	-20.72	-21.95	-38.93	-102.25	18,638
46+	-14.35	-42.99	-32.60	-15.85	-19.18	-36.50	-32.29	-88.14	6,080
Workplace									
Remote	-13.23	-50.79	-33.95	-33.63	-24.78	-32.30	-43.22	-104.88	17,934
On site	-8.59	-42.89	-23.66	-17.15	-13.47	-14.89	-38.94	-97.82	17,120
Region									
Lisbon	-11.30	-47.85	-33.78	-28.86	-28.50	-28.20	-38.72	-100.90	8,192
Porto	-10.45	-45.99	-33.37	-25.72	-16.11	-25.94	-40.37	-103.41	14,672
Other	-10.50	-46.76	-20.29	-22.20	-16.64	-17.62	-43.05	-98.77	12,190

Children									
Only <6	-3.16	-39.11	-23.52	-23.51	-14.92	-7.72	-31.24	-89.56	4,560
<6 & >6	-2.70	-29.56	-26.24	-27.23	-32.46	-47.52	-26.17	-77.64	1,616
Only >6	-10.26	-50.35	-31.71	-34.01	-22.69	-28.81	-46.81	-104.30	7,136
No children	-10.89	-46.53	-27.84	-22.43	-18.03	-22.25	-41.57	-105.32	20,592
Education									
No college degree	-14.47	-47.33	-24.10	-25.14	-14.65	-17.59	-40.64	-95.94	9,264
Bachelors	-13.52	-49.22	-24.41	-24.59	-17.83	-22.69	-39.79	-101.64	11,376
Master+	-6.30	-44.00	-35.55	-26.13	-23.16	-27.87	-41.62	-103.70	14,302
Household equivalent income									
< €750	-8.61	-40.24	-22.35	-17.55	-17.12	-15.77	-34.43	-99.75	7,952
€751 - €1,250	-11.88	-52.70	-21.53	-20.96	-8.37	-15.41	-36.86	-94.28	7,792
€1,251-€2,000	-7.34	-43.71	-38.62	-33.16	-27.50	-30.67	-41.75	-97.91	8,240
>€2,001	-11.66	-47.20	-32.47	-26.72	-21.15	-31.82	-47.41	-110.29	8,286
Student									
Yes	-13.96	-53.32	-29.34	-32.95	-20.37	-23.61	-45.60	-101.90	9,920
No	-9.54	-43.98	-28.78	-22.06	-19.00	-23.42	-39.25	-100.57	25,134

Table S4. Coefficients from the conditional logit model with deaths modelled as a continuous attribute - Subgroups.

	Deaths	Income lost		Education lost		Life Restrictions		Poverty		N
	cont.	2	3	2	3	2	3	2	3	
Gender										
Female	-0.011	-0.078	-0.486	-0.288	-0.206	-0.142	-0.096	-0.450	-1.148	22,064
Male	-0.009	-0.156	-0.506	-0.320	-0.368	-0.290	-0.489	-0.391	-0.928	12,901
Age group										
<25	-0.011	-0.164	-0.585	-0.310	-0.358	-0.194	-0.211	-0.544	-1.163	10,336
26-45	-0.010	-0.073	-0.459	-0.296	-0.252	-0.216	-0.229	-0.406	-1.067	18,638
46+	-0.009	-0.134	-0.403	-0.305	-0.148	-0.180	-0.342	-0.303	-0.826	6,080
Workplace										
Remote	-0.010	-0.133	-0.509	-0.340	-0.337	-0.248	-0.324	-0.433	-1.051	17,934

On site	-0.011	-0.093	-0.463	-0.256	-0.185	-0.146	-0.161	-0.421	-1.057	17,120
Region										
Lisbon	-0.010	-0.114	-0.484	-0.342	-0.292	-0.288	-0.285	-0.392	-1.020	8,192
Oporto	-0.011	-0.112	-0.491	-0.356	-0.275	-0.172	-0.277	-0.431	-1.105	14,672
Other	-0.010	-0.107	-0.478	-0.207	-0.227	-0.170	-0.180	-0.440	-1.009	12,190
Children										
Only <6	-0.012	-0.036	-0.452	-0.272	-0.272	-0.172	-0.089	-0.361	-1.034	4,560
<6 & >6	-0.010	-0.028	-0.302	-0.268	-0.278	-0.332	-0.486	-0.268	-0.794	1,616
Only >6	-0.010	-0.101	-0.495	-0.312	-0.334	-0.223	-0.283	-0.460	-1.026	7,136
No children	-0.011	-0.115	-0.490	-0.293	-0.236	-0.190	-0.234	-0.438	-1.109	20,592
Education										
No college degree	-0.010	-0.150	-0.491	-0.250	-0.261	-0.152	-0.183	-0.422	-0.996	9,264
Bachelors	-0.010	-0.138	-0.502	-0.249	-0.251	-0.182	-0.231	-0.406	-1.036	11,376
Master+	-0.011	-0.067	-0.465	-0.376	-0.276	-0.245	-0.295	-0.440	-1.096	14,302
Household equivalent income										
< €750	-0.010	-0.090	-0.422	-0.235	-0.184	-0.180	-0.165	-0.361	-1.047	7,952
€751 - €1,250	-0.011	-0.129	-0.572	-0.234	-0.228	-0.091	-0.167	-0.400	-1.023	7,792
€1,251-€2,000	-0.010	-0.077	-0.458	-0.405	-0.347	-0.288	-0.321	-0.437	-1.026	8,240
>€2,001	-0.010	-0.118	-0.477	-0.328	-0.270	-0.214	-0.321	-0.479	-1.113	8,286
Student										
Yes	-0.011	-0.152	-0.582	-0.320	-0.359	-0.222	-0.258	-0.498	-1.112	9,920
No	-0.010	-0.097	-0.448	-0.293	-0.225	-0.194	-0.239	-0.400	-1.025	25,134
